# Impact of increasing CD4 count threshold eligibility for antiretroviral therapy initiation on advanced HIV disease and tuberculosis prevalence and incidence in South Africa: an interrupted time series analysis

**DOI:** 10.1136/bmjgh-2024-016631

**Published:** 2025-04-09

**Authors:** Kwabena Asare, Lara Lewis, Johan van der Molen, Yukteshwar Sookrajh, Thokozani Khubone, Thulani Ngwenya, Ntokozo Siyabonga Mkhize, Richard John Lessells, Kogieleum Naidoo, Phelelani Sosibo, Christian Bottomley, Nigel Garrett, Jienchi Dorward

**Affiliations:** 1 Centre for the AIDS Programme of Research in South Africa (CAPRISA), Durban, KwaZulu-Natal, South Africa; 2 Department of Non-Communicable Disease Epidemiology, London School of Hygiene & Tropical Medicine, Faculty of Epidemiology and Population Health, London, UK; 3 eThekwini Municipality Health Unit, eThekwini Municipality, Durban, KwaZulu-Natal, South Africa; 4 Bethesda Hospital, Ubombo, Umkhanyakude District, KwaZulu-Natal, South Africa; 5 KwaZulu-Natal Research and Innovation Sequencing Platform (KRISP), University of KwaZulu-Natal, Durban, KwaZulu-Natal, South Africa; 6 Discipline of Public Health Medicine, School of Nursing and Public Health, University of KwaZulu-Natal, Durban, KwaZulu-Natal, South Africa; 7 South African Medical Research Council (SAMRC)-CAPRISA-TB-HIV Pathogenesis and Treatment Research Unit, Nelson R Mandela School of Medicine, University of KwaZulu-Natal, Durban, KwaZulu-Natal, South Africa; 8 Department of Infectious Disease Epidemiology, London School of Hygiene and Tropical Medicine, London, UK; 9 Nuffield Department of Primary Care Health Sciences, University of Oxford, Oxfordshire, UK

**Keywords:** HIV, tuberculosis, treatment

## Abstract

**Introduction:**

We investigated the impact of increasing CD4 count eligibility for antiretroviral therapy (ART) initiation on advanced HIV disease (AHD) and tuberculosis (TB) prevalence and incidence among people living with HIV (PLHIV) in South Africa.

**Methods:**

We conducted an interrupted time series analysis with de-identified data of PLHIV aged ≥15 years initiating ART between April 2012 and February 2020 at 65 primary healthcare clinics in KwaZulu-Natal, South Africa. Outcomes included monthly proportions of new ART initiators presenting with AHD (CD4 count <200 cells/µL) and TB disease. We created a cohort of monthly ART initiators without TB and evaluated the cumulative incidence of TB within 12 months follow-up. We used segmented binomial regression models to estimate relative risks (RR) of outcomes, allowing for a step and slope change after expanding the ART initiation CD4 count eligibility from <350 to <500 cells/µL in January 2015 and following Universal Test and Treat (UTT) implementation in September 2016.

**Results:**

Among 209 984 participants, median age was 32 (range: 26–38), and 141 499 (67.4%) were female. After January 2015, the risk of AHD at initiation decreased in step by 25.0% (RR=0.750, 95% CI 0.688 to 0.812) and further reduced by 26.9% following UTT implementation (RR=0.731, 95% CI 0.681 to 0.781). The risk of TB at initiation also decreased in step by 27.6% after January 2015 (RR=0.724, 95% CI 0.651 to 0.797) and further decreased by 17.4% after UTT implementation (RR=0.826, 95% CI 0.711 to 0.941) but remained stable among initiators with AHD. Among the incidence cohort, we saw a step decrease in the risk of new TB by 31.4% (RR=0.686, 95% CI 0.465 to 0.907) following UTT implementation. Among the incidence cohort with AHD, there was weak evidence of a step decrease in the risk of new TB (RR=0.755, 95% CI 0.489 to 1.021), but the slope decreased by 9.7% per month (RR=0.903, 95% CI 0.872 to 0.934) following UTT implementation.

**Conclusions:**

Our data support the added benefit of decreased TB co-burden with expanded ART access. Early diagnosis and immediate linkage to care should be prioritised among PLHIV.

**WHAT IS ALREADY KNOWN ON THIS TOPIC**

- Widening access to antiretroviral therapy (ART) for people living with HIV by increasing the CD4 count eligibility from <350 to <500 cells/µL in January 2015 and implementing the Universal Test and Treat (UTT) policy in September 2016, has successfully increased the number of people receiving ART; however, the impacts on advanced HIV disease and tuberculosis co-infection are less well understood.

**WHAT THIS STUDY ADDS**

- After each policy change, we saw a step and slope decline in the prevalence of advanced HIV and tuberculosis disease among new ART initiators, but about a quarter of new initiators consistently presented with advanced HIV disease in the current UTT era.
- The 1-year tuberculosis incidence proportion among new ART initiators decreased in step and slope after UTT implementation.

**HOW THIS STUDY MIGHT AFFECT RESEARCH, PRACTICE OR POLICY**

- The results support the need to upscale UTT implementation with interventions effective for early HIV detection and immediate linkage to care.

## Introduction

 During the HIV epidemic, there has been a gradual expansion of the CD4 count eligibility criteria for initiating people living with HIV (PLHIV) on antiretroviral therapy (ART) in South Africa. Before August 2011, the initiation CD4 count eligibility was <200 cells/µL.^1^ From August 2011, it was expanded to <350 cells/µL and then to <500 cells/µL from January 2015.^1^ In September 2016, WHO’s Universal Test and Treat (UTT) policy was implemented in South Africa, allowing rapid same-day ART initiations regardless of CD4 count level or clinical stage of HIV disease.^2 3^ Likewise, from April 2012, even before UTT, all PLHIV with active tuberculosis (TB) disease in South Africa became eligible for ART initiation, irrespective of the CD4 count level.^4^ These policy changes were informed by evidence of the efficacy of early ART initiation on viral suppression^5^ and the efficacy of combining ART with TB treatment in reducing all-cause mortality.^6^

After implementing these policies, access to ART increased and a higher proportion of PLHIV in South Africa and other low- and middle-income countries (LMICs) initiated ART at higher CD4 counts.^7^,^9^ Consequently, a higher proportion of PLHIV have achieved viral suppression,^10 11^ and population-level HIV incidence has reduced.^12^ The life expectancy of PLHIV has also improved,^13^ and the burden of opportunistic co-infections such as TB has reduced.^14^ These positive health outcomes are likely due to more PLHIV starting ART earlier at higher CD4 counts due to the expansion of initiation CD4 count eligibility over time. A number of cohort and cross-sectional studies have demonstrated the positive health-related impacts of expanded ART access^914^,^18^ in LMICs, but there is limited evidence on the stepwise impact of ART eligibility expansion on trends in advanced HIV disease and the risk of common causes of death, such as TB disease in PLHIV initiating and receiving ART.

We aimed to determine the impact of increasing the ART initiation CD4 count eligibility from <350 to <500 cells/µL in January 2015, and then to UTT implementation in September 2016, on trends in advanced HIV disease at ART initiation, TB prevalence at ART initiation and cumulative TB incidence within 12 months of follow-up. We aimed to evaluate these objectives among ART initiators and in a subcohort of ART initiators with advanced HIV disease from April 2012 to February 2020 in South Africa.

## Methods

### Study design and setting

We conducted a segmented interrupted time series analysis with de-identified routinely collected data from 65 public primary healthcare facilities in KwaZulu-Natal, South Africa, from 2012 to 2020. Fifty-six clinics were in the eThekwini District, and the remaining nine fixed and mobile primary care clinics in the rural uMkhanyakude district. Estimated UNAIDS 90-90-90 targets for HIV care in KwaZulu-Natal were 76-70-93 in 2013 and 92-72-91 in South Africa in 2020.^19 20^ Based on prevailing clinical guidelines, ART initiation was predicated by clinical assessment for pregnancy in females, and CD4 count testing and screening for TB disease in all PLHIV.^21 22^ The guidelines recommend screening for TB in all PLHIV and screening for pregnancy in females during the follow-up clinic visits. TB screening at ART initiation and follow-up visits involves assessments for TB symptoms, followed by GeneXpert testing among symptomatic participants.^21^,^23^ Pregnant women are recommended to receive GeneXpert testing at ART initiation and follow-up visits, regardless of TB symptoms, due to the lower sensitivity of the TB symptom screen in pregnant women. TB preventive therapy (TPT) is recommended for participants for whom active TB is reasonably ruled out based on TB symptom screening. Follow-up clinic visits were usually between 1 and 3 months apart and viral load testing was usually performed one to two times yearly.

We selected the study period to start in April 2012, when PLHIV with TB became eligible for ART initiation, regardless of CD4 count, in South Africa.^4^ We chose February 2020 as the study end point to exclude the COVID-19 lockdown period in South Africa, which began in March 2020.^24^

### Data sources and data management

We used data from South Africa’s TIER.Net electronic database, which contains demographic and clinical data on PLHIV receiving ART and TB care in public sector healthcare clinics. The information captured includes ART and TB treatment dates and regimens, laboratory results including CD4 count, clinic visits and TB status at ART initiation and at each follow-up clinic visit.

### Participants

For the main cohort, we included all PLHIV aged ≥15 years newly initiating ART (ie, excluding participants who were transferring from another clinic) at the study clinics from April 2012 to February 2020. Additionally, we analysed an incidence cohort comprising a subgroup of the main cohort who initiated ART without TB from April 2012 to February 2019. For the incidence cohort, the time frame of ART initiation was set to allow a minimum of 365 days of follow-up duration for all participants before the end of the study period in February 2020. Participants entered the incidence cohort at ART initiation and exited at the earliest of (1) outcome onset (ie, status change to ‘on TB treatment’ within 12 months), (2) the end of the 12-month follow-up or (3) being censored within 12 months (lost to follow-up, died or transferred out, as we could not access or link participants to data at other clinics). We also analysed subcohorts of the main and incidence cohorts for each category of age (15–24, 25–34, 35–44, 45–54 and 55+ years), sex (male and female) and advanced HIV disease at initiation.

### Outcomes

The study outcomes were the monthly number of new ART initiators (main cohort), the monthly proportion of new ART initiators with advanced HIV disease (main cohort), the monthly number and proportion of new ART initiators with active TB disease (main cohort) and the number and proportion of new ART initiators developing active TB disease within 12 months of follow-up (incidence cohort).

We estimated the monthly number of new ART initiators by counting the number of new ART initiation dates per month and, among these, the number with TB and the number with advanced HIV disease (ie, having a CD4 count <200 cells/µL). TB at ART initiation was defined as the participant being on TB treatment at the start of ART. We calculated the monthly proportion of new ART initiators with advanced HIV disease as the number of new ART initiators with advanced HIV disease divided by the monthly number of new ART initiators.

Data for TB diagnoses at ART initiation indicated whether participants had TB, No TB or if TB status was unascertained. We calculated the monthly proportion of new ART initiators with TB as the number of new ART initiators with TB divided by the monthly number of new ART initiators, including those with unascertained TB status. Thus, the monthly proportion of new ART initiators with TB includes participants with unascertained TB status and, hence, captures only the proportion of initiators with a known TB status.

Available TB data at each visit during follow-up indicated whether a participant was on TB treatment or not. Some participants who were not on TB treatment had unascertained TB status. Among the incidence cohort, new TB meant that the participant’s status changed from ‘no TB’ at ART initiation to ‘on TB treatment’ within 12 months of follow-up. In the incidence cohort, we counted the monthly number of ART initiators and, of these, the number that developed new TB within 12 months of follow-up. We estimated the proportion of new TB within 12 months as the number of new TB cases divided by the monthly number of ART initiators. Thus, the proportion of monthly ART initiators developing new TB includes participants with unascertained new TB status and, hence, captures only the proportion of initiators with a known new TB status within 12 months of follow-up.

### Statistical analyses

We calculated descriptive summaries of participant demographics, CD4 count at ART initiation and crude summaries of the primary outcomes from the main and incidence cohorts among participants initiating ART in each CD4 count eligibility period. The demographic variables included in the descriptive summaries were age, sex, pregnancy status in females and district settlement type of the clinics (rural uMkhanyakude and urban eThekwini). We included the most recent available CD4 count recorded from before 180 and up to 30 days after ART initiation.

We fitted segmented time series regression models for all outcomes. The time series started from a baseline period of April 2012 to December 2014, when the initiation CD4 count eligibility was <350 cells/µL, and allowed for step and slope changes from January 2015 to capture the impact of expanding the initiation CD4 count eligibility to <500 cells/µL, and from September 2016 to capture the impact of UTT implementation. We conducted linear regression of the monthly number of new ART initiators, the monthly number of new ART initiators with TB and number of monthly new ART initiators developing new TB within 12 months of follow-up, using the Prais-Winsten method to account for serial autocorrelation of the errors.^25^ We performed binomial regression on the monthly proportion of new ART initiators with advanced HIV disease, the monthly proportion of new ART initiators with TB and the proportion of monthly new ART initiators developing new TB within 12 months of follow-up. We used a log link for the binomial regression models and exponentiated the coefficients to estimate relative risks (RR). We also used Newey-West SEs with lags up to 3 to calculate CIs.^26 27^

We included the following predictor variables in each model: a time variable measured in months (ie, the month of ART initiation), dummy variables indicating pre-implementation or post-implementation date of each CD4 count eligibility expansion (ie, January 2015 for expansion to <500 cells/µL and September 2016 for UTT implementation) to capture the step change in outcomes after each date, a variable indicating time in months since January 2015 (zero for the months before) and time in months since September 2016 (zero for the months before) to capture the slope changes in outcomes, and two Fourier terms consisting of two pairs of sine and cosine transformations of a 12-month cycle to adjust for seasonal trends in outcomes.

We plotted the segmented interrupted time series graphs from each regression model. We included the counterfactual scenarios for each model, defined as situations where only the previous ART initiation CD4 count eligibility criteria were implemented. The counterfactuals were estimated by predicting the outcomes from the segmented regression models, using a modified dataset as follows: (A) the pre-post January 2015 and pre-post September 2016 dummy variables=no, and time since January 2015 and time since September 2016 variables=0, to predict the counterfactuals from January 2015 to August 2016 and (B) the pre-post September 2016 dummy variable=no, and time since September 2016 variable=0, to predict the counterfactuals after UTT implementation.

We conducted subcohort analyses of the segmented regression and interrupted time series by age category (15–24, 25–34, 35–44, 45–54 and 55+ years), sex (male and female) and among initiators with advanced HIV disease. We performed all statistical analyses using R V.4.2.2 (R Foundation for Statistical Computing, Vienna, Austria).^28^

### Patients and public involvement

We used de-identified routinely collected data with no contacts to the patients. Hence, patients and public involvement in the design, conduct, reporting or dissemination of our research was not applicable.

## Results

### Characteristics of participants at ART initiation

From April 2012 to February 2020, 209 984 PLHIV aged ≥15 years newly initiated ART at the clinics (table 1). The median age was 32 years (IQR 26–38). In total, 141 499 (67.4%) were female, of whom 30 369 (21.5%) were pregnant at the time of ART initiation. The number and proportion of initiators with missing CD4 count (ie, CD4 count test not done) was 1,815 (3.4%) when the initiation CD4 count eligibility was <350 cells/µL, 2,918 (5.9%) from January 2015 to August 2016 when the initiation CD4 count eligibility was <500 cells/µL and 17,707 (16.5%) from September 2016 to February 2020 during UTT implementation. Among participants with CD4 tests done at ART initiation, the median CD4 count per period was 227 cells/µL (IQR 130–313) from April 2012 to December 2014, when the initiation CD4 count eligibility was <350 cells/µL, 306 cells/µL (IQR 176–425) from January 2015 to August 2016 when the initiation CD4 count eligibility was <500 cells/µL and 368 cells/µL (IQR 211–557) from September 2016 to February 2020 during UTT implementation. Among all participants, the number and proportion of initiators with a known CD4 count <200 cells/µL were 21 839 (41.2%) when the initiation CD4 count eligibility was <350 cells/µL, 13 700 (27.5%) when the initiation CD4 count eligibility was <500 cells/µL and 20 830 (19.5%) during UTT implementation. The number and proportion of initiators with known TB were 8015 (15.1%) when the initiation CD4 count eligibility was <350 cells/µL, 5465 (11.0%) when the initiation CD4 count eligibility was <500 cells/µL and 7975 (7.4%) during UTT implementation. Among initiators with advanced HIV disease, the number and proportion with known TB were 4538 (20.8%) when the initiation CD4 count eligibility was <350 cells/µL, 2875 (21.0%) when the initiation CD4 count eligibility was <500 cells/µL and 3839 (18.4%) during UTT implementation.

**Table 1:** Characteristics of the main and incidence cohorts

a. Characteristics of the main cohort and TB prevalence at ART initiation				
		Time-period and CD4 count eligibility for ART initiation		
Characteristic	Overall,n=2 09 984	Apr-2012 to Dec-2014 (<350),n=53 044	Jan-2015 to Aug-2016 (<500),n=49 848	Sep-2016 to Feb 2020 (UTT),n=1 07 092
Age in years	32 (26–38)	32 (27–39)	32 (27–38)	31 (26–38)
15–24	35 446 (16.9%)	7379 (13.9%)	8194 (16.4%)	19 873 (18.6%)
25–34	95 885 (45.7%)	23 767 (44.8%)	23 193 (46.5%)	48 925 (45.7%)
35–44	53 695 (25.6%)	14 714 (27.7%)	12 574 (25.2%)	26 407 (24.7%)
45–54	18 641 (8.9%)	5434 (10.2%)	4455 (8.9%)	8752 (8.2%)
55+	6317 (3.0%)	1750 (3.3%)	1432 (2.9%)	3135 (2.9%)
Gender				
Male	68 485 (32.6%)	17 251 (32.5%)	15 848 (31.8%)	35 386 (33.0%)
Female	141 499 (67.4%)	35 793 (67.5%)	34 000 (68.2%)	71 706 (67.0%)
Known to be pregnant (females only)	30 369 (21.5%)	7643 (21.4%)	8924 (26.2%)	13 802 (19.2%)
District				
Rural	7772 (3.7%)	2685 (5.1%)	1862 (3.7%)	3225 (3.0%)
Urban	202 212 (96.3%)	50 359 (94.9%)	47 986 (96.3%)	103 867 (97.0%)
Missing recent CD4 count at ART initiation*	22 440 (10.7%)	1815 (3.4%)	2918 (5.9%)	17 707 (16.5%)
Median recent CD4 count at ART initiation (cells/µl)*	299 (172–453)	227 (130–313)	306 (176–425)	368 (211–557)
Median days since recent CD4 count at ART initiation*	0 (0–5)	0 (0–9)	0 (0–11)	0 (0–0)
Recent CD4 count at ART initiation<200 cells/µl(participants with CD4 test done)*	56 369 (30.1%)	21 839 (42.6%)	13 700 (29.2%)	20 830 (23.3%)
Known recent CD4 count at ART initiation<200 cells/µl*	56 369 (26.8%)	21 839 (41.2%)	13 700 (27.5%)	20 830 (19.5%)
Known TB status at ART initiation‡				
TB	21 455 (10.2%)	8015 (15.1%)	5465 (11.0%)	7975 (7.4%)
No TB	118 598 (56.5%)	31 066 (58.6%)	30 927 (62.0%)	56 605 (52.9%)
No symptoms	63 732 (30.4%)	10 236 (19.3%)	11 685 (23.4%)	41 811 (39.0%)
Symptoms present, sputum test done	517 (0.2%)	226 (0.4%)	115 (0.2%)	176 (0.2%)
Symptoms present, sputum test not done	2 (0.0%)	0 (0.0%)	1 (0.0%)	1 (0.0%)
Symptom screening not done	3700 (1.8%)	2453 (4.6%)	869 (1.7%)	378 (0.4%)
Screening status unknown	1979 (0.9%)	1047 (2.0%)	786 (1.6%)	146 (0.1%)
Known TB status at ART initiation (participants with initiation CD4 count<200 cells/µl)‡				
TB	11 252 (20.0%)	4538 (20.8%)	2875 (21.0%)	3839 (18.4%)
No TB	29 760 (52.8%)	12 235 (56.0%)	7672 (56.0%)	9853 (47.3%)
No symptoms	13 480 (23.9%)	3703 (17.0%)	2770 (20.2%)	7007 (33.6%)
Symptoms present, sputum test done	153 (0.3%)	97 (0.4%)	24 (0.2%)	32 (0.2%)
Symptoms present, sputum test not done	0 (0.0%)	0 (0.0%)	0 (0.0%)	0 (0.0%)
Symptom screening not done	1201 (2.1%)	940 (4.3%)	189 (1.4%)	72 (0.3%)
Screening status unknown	523 (0.9%)	326 (1.5%)	170 (1.2%)	27 (0.1%)

b. Characteristics of the incidence cohort† and TB incidence within 12 months follow-up				
		Time-period and CD4 count eligibility for ART initiation		
Characteristic	Overall,n=1 09 994	Apr-2012 to Dec-2014 (<350),n=31 066	Jan-2015 to Aug-2016 (<500),n=30 927	Sep-2016 to Feb 2019 (UTT),n=48 001
Age in years	31 (26–38)	32 (27–39)	31 (26–38)	31 (26–37)
15–24	18 569 (16.9%)	4236 (13.6%)	5211 (16.8%)	9122 (19.0%)
25–34	50 925 (46.3%)	14 073 (45.3%)	14 540 (47.0%)	22 312 (46.5%)
35–44	27 655 (25.1%)	8543 (27.5%)	7593 (24.6%)	11 519 (24.0%)
45–54	9574 (8.7%)	3134 (10.1%)	2715 (8.8%)	3725 (7.8%)
55+	3271 (3.0%)	1080 (3.5%)	868 (2.8%)	1323 (2.8%)
Gender				
Male	32 730 (29.8%)	9444 (30.4%)	9130 (29.5%)	14 156 (29.5%)
Female	77 264 (70.2%)	21 622 (69.6%)	21 797 (70.5%)	33 845 (70.5%)
Known to be pregnant (females only)	18 264 (23.6%)	4747 (22.0%)	6161 (28.3%)	7356 (21.7%)
District				
Rural	3069 (2.8%)	1303 (4.2%)	577 (1.9%)	1189 (2.5%)
Urban	106 925 (97.2%)	29 763 (95.8%)	30 350 (98.1%)	46 812 (97.5%)
Missing recent CD4 count at ART initiation*	10 542 (9.6%)	1032 (3.3%)	1830 (5.9%)	7680 (16.0%)
Median recent CD4 count at ART initiation (cells/µl)*	300 (181–445)	232 (140–312)	318 (192–431)	380 (225–564)
Median days since recent CD4 count at ART initiation*	0 (0–5)	0 (0–7)	0 (0–9)	0 (0–0)
Recent CD4 count at ART initiation<200 cells/µl (participants with CD4 test done)*	28 384 (28.5%)	12 235 (40.7%)	7672 (26.4%)	8477 (21.0%)
Known recent CD4 count at ART initiation<200 cells/µl*	28 384 (25.8%)	12 235 (39.4%)	7672 (24.8%)	8477 (17.7%)
Number of visits within 12 months after initiation, median (IQR)	7 (4-9)	9 (6-11)	7 (4-8)	7.0 (3.0–9.0)
At least one follow-up clinic visit within the 12 months	101 028 (91.8%)	29 129 (93.8%)	28 534 (92.3%)	43 365 (90.3%)
Screened for TB at least once within 12 months after initiation	98 018 (97.0%)	26 288 (90.2%)	28 382 (99.5%)	43 348 (100.0%)
Number of times screened for TB within 12 months after initiation, median (IQR)	7 (3-10)	7 (2-10)	7 (4-9)	8 (4-10)
Known to have new active TB disease within 12 months after initiation§	1075 (1.0%)	373 (1.2%)	497 (1.6%)	205 (0.4%)
Days to new active TB disease within 12 months after initiation, median (IQR)	86 (51–169)	94 (55–197)	88 (51–169)	79 (36–113)
Known to have new active TB disease within 12 months after initiation (participants with initiation CD4 count<200 cells/µl)§	603 (2.1%)	222 (1.8%)	267 (3.5%)	114 (1.3%)
Days to new active TB disease within 12 months after initiation (participants with initiation CD4 count<200 cells/µl), median (IQR)	84 (49–160)	87 (49–176)	84 (51–160)	78 (33–119)

Data are no. (%) or median (IQR). Percentages may not add up to 100 because of rounding. All percentages were calculated with the total number in the respective column headers as the denominators unless otherwise stated.

* The most recent available CD4 count recorded from before 180 and up to 30 days after ART initiation.

† The incidence cohort involves ART initiators without TB disease from April 2012 to February 2019 to allow 12-month follow-up by the end of the study period in February 2020.

‡ TB at ART initiation was diagnosed by TB symptom screening or GeneXpert testing which ascertained whether a participant had (1) TB, (2) No TB or (3) TB status not ascertained (all other categories).

§ Available TB data during follow-up indicated whether a participant was on TB treatment or TB status was not ascertained.

ART, antiretroviral therapy; IQR, interquartile range; TB, tuberculosis; UTT, Universal Test and Treat.

### Interrupted time series of new ART initiations from April 2012 to February 2020

**Figure 1:**
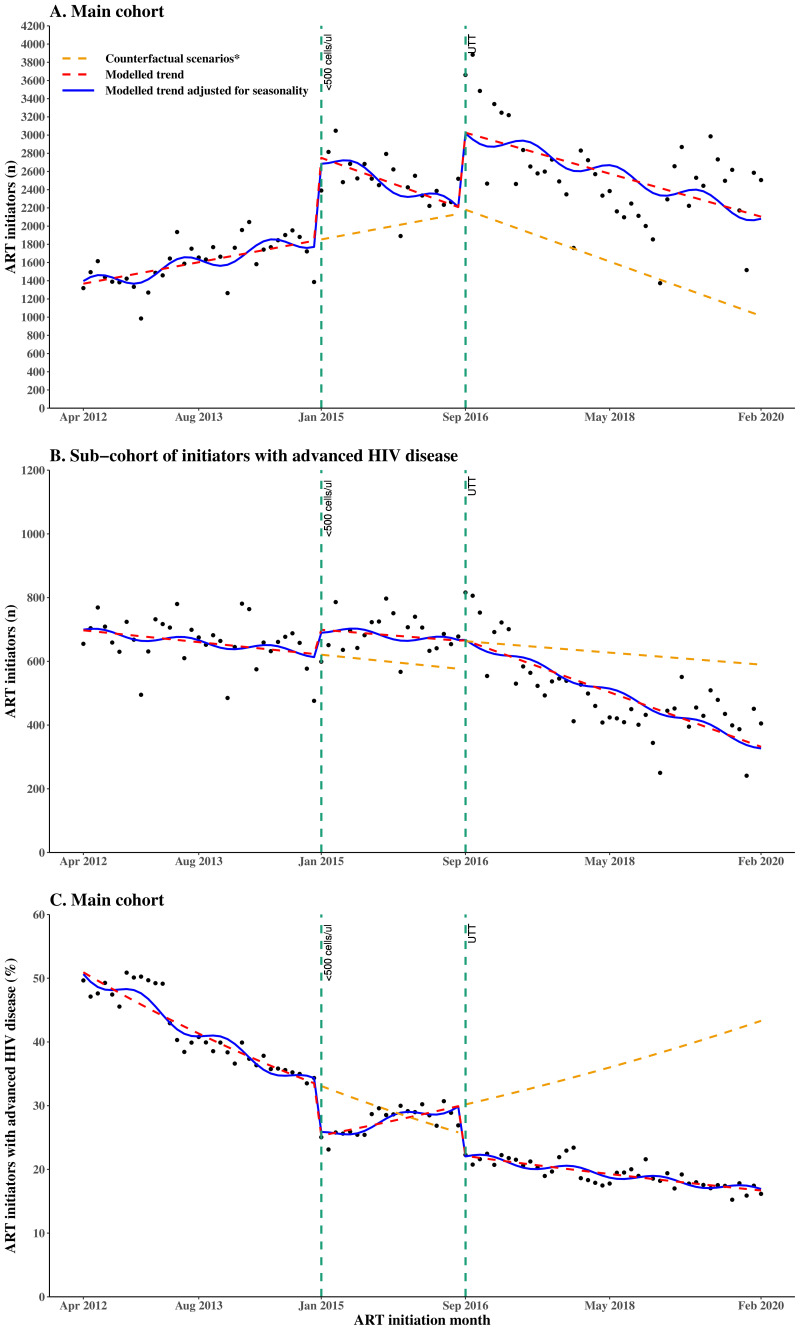
Segmented interrupted time series of (A) monthly number of new ART initiators, (B) monthly number of new ART initiators with known advanced HIV disease and (C) monthly proportion of new ART initiators with known advanced HIV disease. The time series spans three periods of different initiation CD4 count eligibility with two interruptions (vertical dashed lines) indicating the change points. Period 1 is from April 2012 to December 2014, when the initiation CD4 count eligibility was <350 cells/µL. Period 2 is from January 2015 to August 2016, when the initiation CD4 count eligibility was changed to <500 cells/µL. Period 3 is from September 2016 to February 2020 during UTT implementation. *Each counterfactual scenario represents the situation where only the previous initiation CD4 count expansion(s) was implemented. ART, antiretroviral therapy; UTT, Universal Test and Treat.

From the segmented linear regression and interrupted time series analyses (table 2A, figure 1A)**,** from April 2012 to December 2014, when the initiation CD4 count eligibility was <350 cells/µL, the monthly number of ART initiators was gradually rising, although with weak evidence of an increasing trend (coefficient 16.4, 95% CI −0.8, 33.6). With the expansion of the initiation CD4 count eligibility to <500 cells/µL in January 2015, there was a step increase in the monthly number of ART initiators (coefficient 923.8, 95% CI 428.3, 1419.3) and a decrease in slope (coefficient −46.7, 95% CI −86.4, –7.0). From September 2016, when UTT was implemented, there was a further step increase in the monthly number of ART initiators (coefficient 876.3, 95% CI 413.8, 1338.8) with no evidence of a slope change (coefficient 7.9, 95% CI −29.4, 45.2). Among initiators with advanced HIV disease (table 2A, figure 1B), the monthly number of ART initiators was stable over time when the initiation CD4 count eligibility was <350 cells/µL and continued to remain stable with no step change (coefficient 77.2, 95% CI −33.1, 187.5) and no slope change (coefficient 0.0, 95% CI −8.6, 8.6) after the CD4 count eligibility was changed to <500 cells/µL. In September 2016, when UTT was implemented, there was no evidence of a step change (coefficient 14.4, 95% CI −88.1, 116.9), and a non-significant negative slope change (coefficient −6.0, 95% CI −14.1, 2.1), such that the monthly number of ART initiators with AHD decreased slightly after implementation of UTT (coefficient −8.1, 95% CI −10.7, −5.5).

**Table 2:** Impact of increasing CD4 count eligibility threshold for ART initiation on new ART initiations, advanced HIV disease at initiation and active TB disease prevalence at initiation from April 2012 to February 2020

	Time period and CD4 count eligibility for ART initiation						
(A) Linear regression* of the monthly number of new ART initiators	April 2012 to December 2014(<350 cells/µL)	January 2015 to August 2016(<500 cells/µL)			September 2016 to February 2020(UTT)		
Cohort	Baseline slopeCoefficient (95% CI)	Step changeCoefficient (95% CI)	Slope changeCoefficient (95% CI)	New slopeCoefficient (95% CI)	Step changeCoefficient (95% CI)	Slope changeCoefficient (95% CI)	New slopeCoefficient (95% CI)
Main	16.4 (−0.8, 33.6)	923.8 (428.3, 1419.3)	−46.7 (−86.4,–7.0)	−30.3 (−64.6, 4.0)	876.3 (413.8, 1338.8)	7.9 (−29.4, 45.2)	−22.4 (−34.4, – 10.4)
Females	11.9 (−0.1, 23.9)	671.9 (328.8, 1015.0)	−37.2 (−65.0, –9.4)	−25.3 (−49.1, –1.5)	682.9 (362.3, 1003.5)	6.7 (−19.3, 32.7)	−18.5 (−26.9, – 10.1)
Males	4.5 (-0.8, 9.8)	243.3 (85.4, 401.2)	−8.9 (−21.3, 3.5)	−4.4 (−15.2, 6.4)	185.2 (38.3, 332.1)	0.6 (−11.0, 12.2)	−3.8 (−7.5, – 0.1)
15–24 years	5.5 (2.2, 8.8)	141.7 (49.1, 234.3)	−9.7 (−17.3,–2.1)	−4.2 (−10.7, 2.3)	173.8 (87.1, 260.5)	1.2 (−5.9, 8.3)	−3.0 (−5.3, –0.7)
25–34 years	8.2 (0.8, 15.6)	410.1 (191.2, 629.0)	−18.9 (−36.0, –1.8)	−10.7 (−25.7, 4.3)	362.4 (158.8, 566.0)	−0.2 (−16.3, 15.9)	−10.8 (−15.9, –5.7)
35–44 years	2.7 (−1.5, 6.9)	222.5 (97.6, 347.4)	−10.9 (−20.7, –1.1)	−8.2 (−16.7, 0.3)	201.6 (85.5, 317.7)	3 (-6.2, 12.2)	−5.2 (−8.1, –2.3)
45–54 years	−0.1 (−1.9, 1.7)	106.3 (55.6, 157.0)	−4.8 (−8.9, – 0.7)	−4.8 (−8.3, –1.3)	91.4 (44.0, 138.8)	2.3 (-1.6, 6.2)	−2.5 (-3.8, –1.2)
55+years	0.0 (-0.8, 0.8)	36.1 (12.6, 59.6)	−1.5 (−3.4, 0.4)	−1.6 (−3.2, 0.0)	36.2 (14.3, 58.1)	0.8 (−1.0, 2.6)	−0.8 (−1.4, –0.2)
ART initiation CD4 count <200 cells/µL (participants with CD4 test done)	−2.1 (−5.8, 1.6)	77.2 (−33.1, 187.5)	0.0 (−8.6, 8.6)	−2.1 (−9.6, 5.4)	14.4 (−88.1, 116.9)	−6 (−14.1, 2.1)	−8.1 (−10.7, –5.5)

(B) Binomial regression† of the monthly proportion of new ART initiators with known CD4 count <200 cells/µL	April 2012 to December 2014(<350 cells/µL)	January 2015 to August 2016(<500 cells/µL)			September 2016 to February 2020(UTT)		
Cohort	Baseline slopeRR (95% CI)	Step changeRR (95% CI)	Slope changeRR (95% CI)	New slopeCoefficient (95% CI)	Step changeRR (95% CI)	Slope changeRR (95% CI)	New slopeCoefficient (95% CI)
Main	0.986 (0.984, 0.988)	0.750 (0.688, 0.812)	1.023 (1.018, 1.028)	0.010 (0.006, 0.014)	0.731 (0.681, 0.781)	0.984 (0.979, 0.989)	−0.007 (−0.008, –0.006)
Females	0.981 (0.979, 0.983)	0.758 (0.668, 0.848)	1.029 (1.022, 1.036)	0.009 (0.003, 0.015)	0.710 (0.631, 0.789)	0.984 (0.977, 0.991)	−0.007 (−0.008, –0.006)
Males	0.994 (0.993, 0.995)	0.761 (0.725, 0.797)	1.012 (1.009, 1.015)	0.007 (0.004, 0.010)	0.798 (0.755, 0.841)	0.985 (0.981, 0.989)	-0.009 (-0.011, -0.007)
15–24 years	0.970 (0.965, 0.975)	0.845 (0.732, 0.958)	1.039 (1.032, 1.046)	0.008 (0.002, 0.014)	0.745 (0.646, 0.844)	0.981 (0.974, 0.988)	−0.011 (−0.014, – 0.008)
25–34 years	0.985 (0.983, 0.987)	0.752 (0.669, 0.835)	1.027 (1.021, 1.033)	0.011 (0.005, 0.017)	0.741 (0.668, 0.814)	0.981 (0.975, 0.987)	−0.008 (−0.009, –0.007)
35–44 years	0.992 (0.990, 0.994)	0.758 (0.681, 0.835)	1.016 (1.009, 1.023)	0.008 (0.002, 0.014)	0.750 (0.682, 0.818)	0.987 (0.980, 0.994)	−0.005 (−0.007, –0.003)
45–54 years	0.997 (0.994, 1.000)	0.685 (0.618, 0.752)	1.013 (1.008, 1.018)	0.010 (0.006, 0.014)	0.660 (0.550, 0.770)	0.990 (0.985, 0.995)	0.000 (-0.004, 0.004)
55+ years	0.992 (0.989, 0.995)	0.628 (0.519, 0.737)	1.029 (1.020, 1.038)	0.020 (0.012, 0.028)	0.724 (0.589, 0.859)	0.976 (0.967, 0.985)	−0.004 (−0.009, 0.001)

(C) Linear regression* of the monthly number of new ART initiators with known TB	April 2012 to December 2014(<350 cells/µL)	January 2015 to August 2016(<500 cells/µL)			September 2016 to February 2020(UTT)		
Cohort	Baseline slopeCoefficient (95% CI)	Step changeCoefficient (95% CI)	Slope changeCoefficient (95% CI)	New slopeCoefficient (95% CI)	Step changeCoefficient (95% CI)	Slope changeCoefficient (95% CI)	New slopeCoefficient (95% CI)
Main	2.5 (1.4, 3.6)	22.5 (−12.9, 57.9)	−5.3 (−8.0, –2.6)	−2.8 (−5.2, −0.4)	28.1 (−4.7, 60.9)	−1.2 (−3.7, 1.3)	−4.0 (-4.8, −3.2)
Females	0.8 (0.1, 1.5)	19.2 (−0.8, 39.2)	−3.1 (−4.7, –1.5)	−2.3 (−3.7, –0.9)	20.6 (2.0, 39.2)	0.2 (−1.3, 1.7)	−2.2 (-2.7,−1.7)
Males	1.7 (1.0, 2.4)	2.7 (−18.0, 23.4)	−2.1 (-3.7, -0.5)	-0.4 (-1.8, 1.0)	6.7 (−12.5, 25.9)	−1.4 (−2.9, 0.1)	−1.9 (−2.4, –1.4)
15–24 years	0.4 (0.2, 0.6)	0.7 (−6.2, 7.6)	-0.7 (-1.2, -0.2)	-0.3 (-0.8, 0.2)	6.7 (0.4, 13.0)	−0.3 (−0.8, 0.2)	−0.5 (−0.7, −0.3)
25–34 years	1 (0.5, 1.5)	8.8 (−6.2, 23.8)	-1.6 (-2.7, -0.5)	−0.6 (−1.6, 0.4)	5.8 (−8.1, 19.7)	−1.3 (−2.4, –0.2)	−1.9 (−2.2, –1.6)
35–44 years	0.9 (0.5, 1.3)	7.2 (−5.2, 19.6)	−2.2 (−3.1, –1.3)	−1.3 (−2.1, –0.5)	8.4 (−3.0, 19.8)	0.2 (−0.7, 1.1)	−1.1 (−1.4, –0.8)
45–54 years	0.2 (−0.0, 0.4)	2.2 (−5.3, 9.7)	−0.6 (−1.2, –0.0)	−0.4 (−0.9, 0.1)	3.6 (−3.3, 10.5)	0.1 (−0.4, 0.6)	−0.4 (−0.6, –0.2)
55+ years	0.1 (−0.0, 0.2)	0.3 (−3.5, 4.1)	−0.1 (−0.4, 0.2)	−0.1 (−0.4, 0.2)	2.8 (−0.7, 6.3)	−0.1 (−0.4, 0.2)	−0.2 (−0.3, –0.1)
ART initiation CD4 count <200 cells/µL (participants with CD4 test done)	0.1 (−0.6, 0.8)	15.2 (v5.6, 36.0)	−0.9 (−2.5, 0.7)	−0.8 (−2.2, 0.6)	4.6 (−14.6, 23.8)	−1.5 (−3.0, –0.0)	−2.4 (−2.9, –1.9)

(D) Binomial regression† of the monthly proportion of new ART initiators with known TB	April 2012 to December 2014(<350 cells/µL)	January 2015 to August 2016(<500 cells/µL)			September 2016 to February 2020(UTT)		
Cohort	Baseline slopeCoefficient (95% CI)	Step changeRR (95% CI)	Slope changeRR (95% CI)	New slopeCoefficient (95% CI)	Step changeRR (95% CI)	Slope changeRR (95% CI)	New slopeCoefficient (95% CI)
Main	1.000 (0.996, 1.004)	0.724 (0.651, 0.797)	1.001 (0.996, 1.006)	0.001 (−0.003, 0.005)	0.826 (0.711, 0.941)	0.988 (0.981, 0.995)	−0.011 (−0.017, –0.005)
Females	0.996 (0.991, 1.001)	0.752 (0.657, 0.847)	1.000 (0.993, 1.007)	−0.004 (−0.009, 0.001)	0.850 (0.745, 0.955)	0.989 (0.982, 0.996)	−0.015 (−0.020, –0.010)
Males	1.006 (1.004, 1.008)	0.728 (0.659, 0.797)	0.995 (0.990, 1.000)	0.001 (−0.003, 0.005)	0.870 (0.735, 1.005)	0.988 (0.980, 0.996)	−0.011 (−0.018, –0.004)
15–24 years	0.992 (0.985, 0.999)	0.731 (0.556, 0.906)	1.005 (0.992, 1.018)	−0.002 (−0.011, 0.007)	0.952 (0.801, 1.103)	0.980 (0.968, 0.992)	−0.022 (−0.029, –0.015)
25–34 years	0.998 (0.995, 1.001)	0.730 (0.661, 0.799)	1.005 (0.999, 1.011)	0.003 (-0.002, 0.008)	0.811 (0.687, 0.935)	0.983 (0.974, 0.992)	−0.014 (−0.020, –0.008)
35–44 years	1.005 (1.002, 1.008)	0.749 (0.687, 0.811)	0.992 (0.988, 0.996)	−0.003 (−0.006, 0.000)	0.830 (0.695, 0.965)	0.995 (0.989, 1.001)	−0.008 (−0.014, –0.002)
45–54 years	1.006 (0.999, 1.013)	0.645 (0.415, 0.875)	1.000 (0.987, 1.013)	0.006 (−0.006, 0.018)	0.783 (0.598, 0.968)	0.991 (0.977, 1.005)	−0.003 (−0.009, 0.003)
55+ years	1.010 (0.999, 1.021)	0.630 (0.339, 0.921)	1.002 (0.980, 1.024)	0.011 (−0.008, 0.030)	0.881 (0.512, 1.250)	0.980 (0.956, 1.004)	−0.009 (−0.023, 0.005)
ART initiation CD4 count <200 cells/µL (participants with CD4 test done)	1.004 (1.002, 1.006)	0.996 (0.912, 1.080)	0.992 (0.987, 0.997)	−0.004 (−0.009, 0.001)	1.061 (0.947, 1.175)	0.995 (0.989, 1.001)	−0.009 (−0.013,–0.005)

Each row presents results from the analysis among participants in the stated cohort. The proportion of known TB cases at ART initiation was calculated as the number of TB cases divided by the number of initiators. All step and slope changes in estimates (coefficients and RRs) are in reference to the end of the previous policy implementation date.

* Prais-Winsten method was used to account for serial autocorrelation of the error terms.

† Newey-West SEs with lag up to three were used to calculate CI.

ART, antiretroviral therapy; RR, relative risk; TB, tuberculosis; UTT, Universal Test and Treat.

### Interrupted time series of advanced HIV disease prevalence at ART initiation from April 2012 to February 2020

The segmented binomial regression and interrupted time series analyses of the proportion of initiators with a known status of advanced HIV disease are presented in table 2B and figure 1C. The results show a decreasing trend in the proportion of initiators with advanced HIV disease, declining by 1.4% per month (RR 0.986, 95% CI 0.984, 0.988) from April 2012 to December 2014, when the initiation CD4 count eligibility was <350 cells/µL. Afterwards, when the initiation CD4 count eligibility was expanded to <500 cells/µL in January 2015, there was a step decrease by 25.0% (RR 0.750, 95% CI 0.688, 0.812) and a slope increase by 2.3% (RR 1.023, 95% CI 1.018, 1.028). Subsequently, when UTT was implemented in September 2016, there was a further step decrease by 26.9% (RR 0.731, 95% CI 0.681, 0.781) and a slope decrease of 1.6% (RR 0.984, 95% CI 0.979, 0.989). The differences in step and slope changes in the proportion of initiators with known advanced HIV disease were similar by sex and by age group following the expansion of the initiation CD4 count eligibility from <350 to <500 cells/µL and following the implementation of UTT.

### Interrupted time series of active TB disease prevalence at ART initiation from April 2012 to February 2020

**Figure 2:**
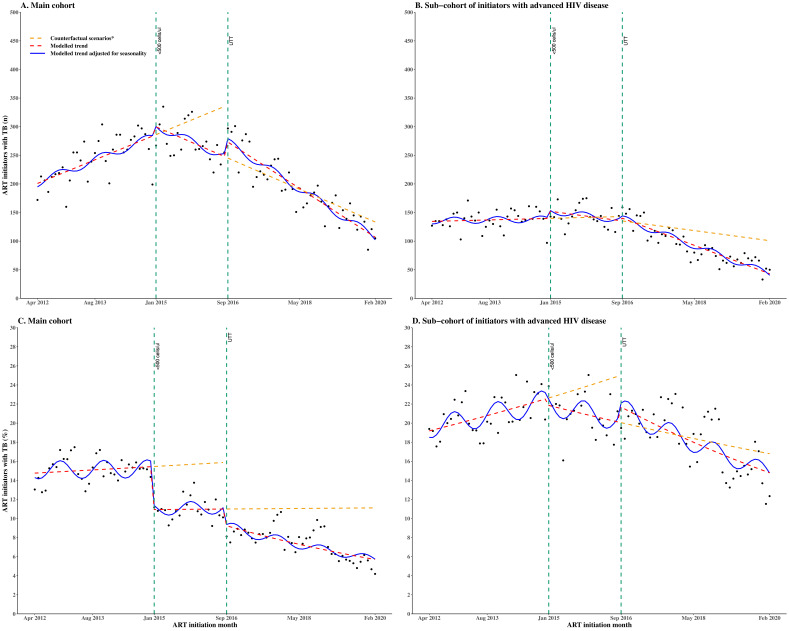
Segmented interrupted time series of (A) monthly number of new ART initiators with known TB disease, (B) monthly number of new ART initiators with advanced HIV with known TB disease, (C) monthly proportion of new ART initiators with known TB disease, (D) monthly proportion of new ART initiators with advanced HIV with known TB disease. The time series spans three periods of different initiation CD4 count eligibility with two interruptions (vertical dashed lines) indicating the change points. Period 1 is from April 2012 to December 2014, when the initiation CD4 count eligibility was <350 cells/µL. Period 2 is from January 2015 to August 2016, when the initiation CD4 count eligibility was changed to <500 cells/µL. Period 3 is from September 2016 to February 2020 during UTT implementation. *Each counterfactual scenario represents the situation where only the previous initiation CD4 count expansion was implemented. ART, antiretroviral therapy; TB, tuberculosis; UTT, Universal Test and Treat.

From April 2012 to December 2014, when the initiation CD4 count eligibility was <350 cells/µL, the monthly number of initiators with known TB was gradually rising (table 2C, figure 2A). There was no evidence of a step change in January 2015, when the initiation CD4 count eligibility was increased to <500 cells/µL, but the slope decreased. After the introduction of UTT, there were no observed changes in the step or slope of the monthly number of initiators with known TB. Among initiators with advanced HIV disease (table 2C, figure 2B), the monthly number of initiators with known TB was stable when the initiation CD4 count eligibility was <350 cells/µL. Afterwards, there was no evidence of a step change and no evidence of a slope change following the expansion of the initiation CD4 count eligibility to <500 cells/µL in January 2015. After UTT was implemented, there was no evidence of a step change, but weak evidence of a slope decrease (coefficient −1.5, 95% CI −3.0 to –0.0), such that after UTT, the monthly number of AHD initiators with known TB decreased by −2.4 (95% CI −2.9 to −1.9).

The monthly number of initiators with TB is a function of the number of people initiating ART (which changes over time), so we also present analyses of the proportion of initiators with known TB. Table 2D and figure 2C show that the proportion of initiators with known TB was stable when the initiation CD4 count eligibility was <350 cells/µL. It then decreased by 27.6% (RR 0.724, 95% CI 0.651, 0.797) from January 2015, when the initiation CD4 count eligibility was expanded to <500 cells/µL, but the slope remained stable (RR 1.001 95% CI 0.996, 1.006). Following UTT implementation in September 2016, there was a further step decrease by 17.4% (RR 0.826, 95% CI 0.711, 0.941) and a slope decline by 1.2% (RR 0.988 95% CI 0.981, 0.995). The proportion of initiators with TB across age groups and sexes was similar following the initiation CD4 count eligibility expansion from <350 to <500 cells/µL and following UTT implementation. Among initiators with advanced HIV disease (table 2D and figure 2D), the proportion with known TB was stable over time when the initiation CD4 count eligibility was <350 cells/µL. With the expansion of the initiation CD4 count eligibility to <500 cells/µL, there was no evidence of a step change (RR 0.996, 95% CI 0.912, 1.080), but the slope decreased by 0.8% (RR 0.992, 95% CI 0.987, 0.997). Following the implementation of UTT from September 2016, there was no evidence of a step change (RR 1.061, 95% CI 0.947, 1.175), but weak evidence of a slope decrease (RR 0.995, 95% CI 0.989, 1.001).

### Characteristics of the incidence cohort and follow-up outcomes within 12 months after ART initiation

The incidence cohort involved 109 994 participants with no active TB disease at ART initiation from April 2012 to February 2019 . The median age was 31 years (IQR 26–38), and 77 264 (70.2%) were female, of whom 18 264 (23.6%) were pregnant at the time of ART initiation. Among participants with a CD4 test done at ART initiation, the median CD4 count was 300 cells/µL (IQR 181–445), and 28 384 (25.8%) started ART with known advanced HIV disease. The number and proportion with known advanced HIV disease were 12 235 (39.4%) when the initiation CD4 count eligibility was <350 cells/µL, 7672 (24.8%) when the initiation CD4 count eligibility was <500 cells/µL and 8477 (17.7%) during UTT implementation. The incidence cohort made a median of 7 (IQR 4–9) follow-up clinic visits within 12 months, and 101 028 (91.8%) made at least one follow-up clinic visit within the 12 months. Participants attending at least one follow-up clinic visit within 12 months were screened for TB a median of seven times (IQR 3–10), and 98 018 (97.0%) were screened at least once for TB. The number and proportion of participants known to develop new TB within 12 months of follow-up were 373 (1.2%) when the initiation CD4 count eligibility was <350 cells/µL, 497 (1.6%) when the initiation CD4 count eligibility was <500 cells/µL and 205 (0.4%) during UTT implementation. The median days to developing new TB within 12 months of follow-up were 94 (55–197) when the initiation CD4 count eligibility was <350 cells/µL, 88 (51–169) when the initiation CD4 count eligibility was <500 cells/µL and 79 (36–113) during UTT implementation.

### Interrupted time series of the proportion of monthly ART initiators in the incidence cohort developing new TB within 12 months of follow-up

**Figure 3:**
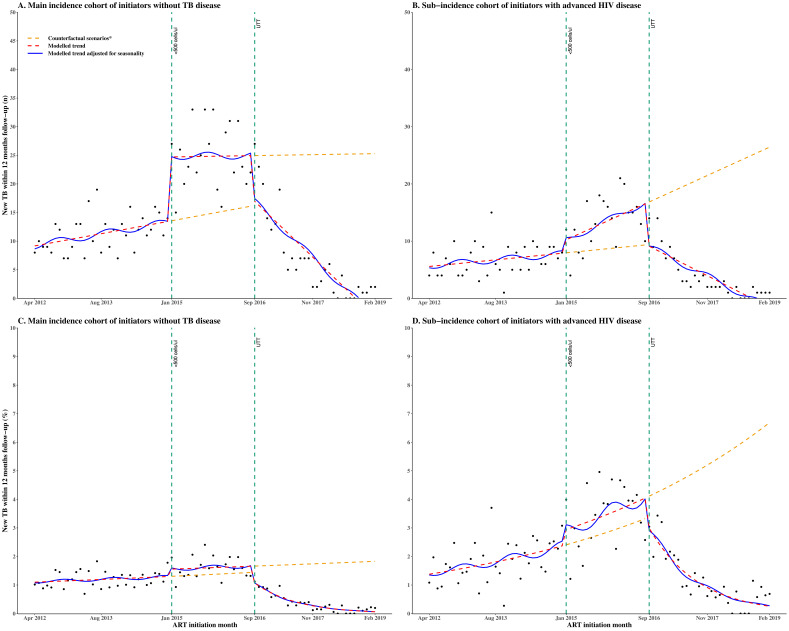
Segmented interrupted time series among the incidence cohort of initiators without TB disease: (A) monthly number known to develop new TB disease within 12 months of follow-up, (B) monthly number with advanced HIV known to develop new TB disease within 12 months of follow-up, (C) monthly proportion known to develop new TB disease within 12 months of follow-up and (D) monthly proportion with advanced HIV known to develop new TB disease within 12 months of follow-up. The time series spans three periods of different initiation CD4 count eligibility with two interruptions (vertical dashed lines) indicating the change points. Period 1 is from April 2012 to December 2014, when the initiation CD4 count eligibility was <350 cells/µL. Period 2 is from January 2015 to August 2016, when the initiation CD4 count eligibility was changed to <500 cells/µL. Period 3 is from September 2016 to February 2019 during UTT implementation. *Each counterfactual scenario represents the situation where only the previous initiation CD4 count expansion was implemented. ART, antiretroviral therapy; TB, tuberculosis; UTT, Universal Test and Treat.

Results from the segmented linear regression and interrupted time series analyses (table 3A and figure 3A) demonstrate no evidence of a changing trend in the number of monthly initiators known to develop new TB within 12 months of follow-up when the initiation CD4 count eligibility was <350 cells/µL. Following the expansion of the initiation CD4 count eligibility to <500 cells/µL in January 2015, there was a step increase but no evidence of a slope change. With the implementation of UTT from September 2016, there was a step decrease and a slope decrease. Among the incidence cohort with advanced HIV disease (table 3A and figure 3B), the number known to develop new TB within 12 months of follow-up exhibited a stable trend when the initiation CD4 count eligibility was <350 cells/µL. Following the expansion of the initiation CD4 count eligibility to <500 cells/µL in January 2015, there was weak evidence of a step increase (coefficient 2.1, 95% CI −1.1, 5.3) and weak evidence of a slope increase (coefficient 0.3, 95% CI 0.1, 0.5). However, following UTT implementation from September 2016, there was a step decrease (coefficient −7.4, 95% CI -10.5, –4.3) and a slope decrease (coefficient −0.7, 95% CI −0.9 to –0.5).

**Table 3:** Impact of increasing CD4 count eligibility threshold for ART initiation on cumulative TB incidence within 12 months of follow-up among the incidence cohort*

	Time period and CD4 count eligibility for ART initiation						
(A) Linear regression† of the number of monthly ART initiators known to develop new TB within 12 months of follow-up	April 2012 to December 2014(<350 cells/µL)	January 2015 to August 2016(<500 cells/µL)			September 2016 to February 2019(UTT)		
Cohort	Baseline slopeCoefficient (95% CI)	Step changeCoefficient (95% CI)	Slope changeCoefficient (95% CI)	New slopeCoefficient (95% CI)	Step changeCoefficient (95% CI)	Slope changeCoefficient (95% CI)	New slopeCoefficient (95% CI)
Main	0.1 (-0.1, 0.3)	11.6 (6.7, 16.5)	−0.1 (−0.5, 0.3)	0.0 (−0.3, 0.3)	−7.4 (−12.2, −2.6)	−0.7 (−1.1, −0.3)	−0.7 (−0.9, −0.5)
Females	0.0 (−0.1, 0.1)	8.8 (5.7, 11.9)	−0.2 (−0.4, 0.0)	−0.1 (−0.3, 0.1)	−3 (−6.1, 0.1)	−0.2 (−0.4, 0.0)	−0.4 (−0.5, −0.3)
Males	0.1 (0.0, 0.2)	2.6 (−0.4, 5.6)	0.1 (−0.1, 0.3)	0.2 (−0.0, 0.4)	−4.8 (−7.8, −1.8)	−0.5 (−0.7, −0.3)	−0.3 (−0.4, −0.2)
15–24 years	0.0 (−0.0, 0.0)	0.9 (-0.3, 2.1)	0.0 (−0.1, 0.1)	0.0 (−0.1, 0.1)	−1.1 (−2.3,0.1)	−0.1 (−0.2, −0.0)	−0.1 (−0.1, −0.1)
25–34 years	0.0 (−0.1, 0.1)	6.4 (3.7, 9.1)	0.0 (−0.2, 0.2)	0.0 (−0.2, 0.2)	−4.5 (−7.1, −1.9)	−0.3 (-0.5, -0.1)	0.3 (−0.4, −0.2)
35–44 years	0.0 (−0.1, 0.1)	1.5 (−1.5, 4.5)	0.0 (−0.2, 0.2)	0.1 (−0.1, 0.3)	−2.6 (−5.6, 0.4)	−0.3 (−0.5, −0.1)	−0.2 (−0.3, −0.1)
45–54 years	0.0 (−0.1, 0.1)	1.9 (0.2, 3.6)	−0.1 (−0.2, 0.0)	−0.1 (−0.2, 0.0)	−0.1 (−1.8, 1.6)	0 (−0.1, 0.1)	−0.1 (−0.2, −0.0)
55+ years	0.0 (−0.0, 0.0)	1.3 (0.6, 2.0)	−0.1 (−0.2, −0.0)	−0.1 (−0.1, −0.1)	0.8 (0.1, 1.5)	0 (−0.1, 0.1)	−0.0 (−0.0, 0.0)
ART initiation CD4 <200 cells/µL (participants with CD4 test done)	0.1 (0.0, 0.2)	2.1 (−1.1, 5.3)	0.3 (0.1, 0.5)	0.3 (0.1, 0.5)	−7.4 (−10.5, −4.3)	−0.7 (−0.9, −0.5)	−0.4 (−0.5, −0.3)

(B) Binomial regression‡ of the proportion of monthly ART initiators known to develop new TB within 12 months of follow-up	April 2012 to December 2014(<350 cells/µL)	January 2015 to August 2016(<500 cells/µL)			September 2016 to February 2019(UTT)		
Cohort	Baseline slopeCoefficient (95% CI)	Step changeRR (95% CI)	Slope changeRR (95% CI)	New slopeCoefficient (95% CI)	Step changeRR (95% CI)	Slope changeRR (95% CI)	New slopeCoefficient (95% CI)
Main	1.004 (0.999, 1.009)	1.215 (1.001, 1.429)	0.999 (0.983, 1.015)	0.004 (−0.011, 0.019)	0.686 (0.465, 0.907)	0.906 (0.882, 0.930)	−0.096 (−0.115, −0.077)
Females	0.998 (0.990, 1.006)	1.481 (1.273, 1.689)	0.996 (0.981, 1.011)	−0.006 (−0.018, 0.006)	0.703 (0.477, 0.929)	0.919 (0.891, 0.947)	−0.090 (−0.115, −0.065)
Males	1.013 (1.003, 1.023)	0.961 (0.571, 1.351)	0.999 (0.968, 1.030)	0.013 (-0.016, 0.042)	0.717 (0.289, 1.145)	0.888 (0.843, 0.933)	−0.106 (−0.142, −0.070)
15–24 years	1.001 (0.953, 1.049)	1.119 (0.160, 2.078)	1.012 (0.948, 1.076)	0.012 (−0.027, 0.051)	0.635 (−0.125, 1.395)	0.895 (0.821, 0.969)	−0.099 (−0.168, −0.030)
25–34 years	1.003 (0.992, 1.014)	1.418 (1.163, 1.673)	0.999 (0.975, 1.023)	0.002 (−0.019, 0.023)	0.696 (0.402, 0.990)	0.895 (0.858, 0.932)	−0.109 (−0.136, −0.082)
35–44 years	1.009 (0.994, 1.024)	0.861 (0.397, 1.325)	1.011 (0.976, 1.046)	0.020 (−0.012, 0.052)	0.585 (0.205, 0.965)	0.897 (0.854, 0.940)	−0.089 (−0.119, −0.059)
45–54 years	1.018 (0.996, 1.040)	1.189 (0.532, 1.846)	0.976 (0.931, 1.021)	−0.006 (−0.043, 0.031)	0.700 (−0.099, 1.499)	0.956 (0.873, 1.039)	−0.051 (−0.124, 0.022)
55+ years	0.981 (0.942, 1.020)	5.139 (4.051, 6.227)	0.912 (0.823, 1.001)	−0.112 (−0.189, −0.035)	5.216 (4.086, 6.346)	0.947 (0.796, 1.098)	−0.166 (−0.297, −0.035)
ART initiation CD4 <200 cells/µL (participants with CD4 test done)	1.016 (1.010, 1.022)	1.220 (0.915, 1.525)	1.003 (0.980, 1.026)	0.019 (−0.003, 0.041)	0.755 (0.489, 1.021)	0.903 (0.872, 0.934)	−0.083 (−0.105, −0.061)

Each row presents results from the analysis among participants in the stated cohort.

All step and slope changes in estimates (coefficients and RRs) are in reference to the end of the previous policy implementation date.

* The incidence cohort involves ART initiators without TB disease from April 2012 to February 2019 to allow 12 months of follow-up by the end of the study period in February 2020.

† Prais-Winsten method was used to account for serial autocorrelation of the error terms.

‡ Newey-West SEs with lag up to three were used to calculate CI.

ART, antiretroviral therapy; RR, relative risk; TB, tuberculosis.

Results from the segmented binomial regression and interrupted time series analyses (table 3B and figure 3C) demonstrate that the proportion of monthly initiators known to develop new TB within 12 months of follow-up was stable when the initiation CD4 count was <350 cells/µL. When the initiation CD4 count was increased to <500 cells/µL in January 2015, there was no evidence of a step change (RR 1.215, 95% CI 1.010, 1.429) and no evidence of a slope change (RR 0.999, 95% CI 0.983, 1.015). However, following the implementation of UTT in September 2016, there was a step decrease by 31.4% (RR 0.686, 95% CI 0.465, 0.907) and a slope decrease of 9.4% (RR 0.906, 95% CI 0.882, 0.930). Among the incidence cohort with advanced HIV disease, the results (table 3B and figure 3D) show no evidence of a changing trend in the proportion of monthly initiators known to develop new TB within 12 months of follow-up when the initiation CD4 count was <350 cells/µL. Subsequently, when the initiation CD4 count eligibility was expanded to <500 cells/µL in January 2015, there was no evidence of a step change (RR 1.220, 95% CI 0.915, 1.525) and no evidence of a slope change (RR 1.003, 95% CI 0.980 to 1.026). However, from September 2016, when UTT was implemented, there was weak evidence of a step change (RR 0.755 95% CI 0.489, 1.021), and the slope decreased by 9.7% (RR 0.903, 95% CI 0.872, 0.934).

## Discussion

We present results from an interrupted time series analysis of de-identified, large-scale routinely collected clinic data from South Africa’s ART programme between April 2012 and February 2020. The findings show that, following the expansion of the ART initiation CD4 count eligibility from <350 to <500 cells/µL in January 2015 and the subsequent implementation of UTT in September 2016, ART initiations immediately increased but thereafter declined, approaching the counterfactual levels associated with the previous CD4 count eligibility criteria. The declining trend in new ART initiations was partly due to the existing backlog of ART-naïve PLHIV who immediately became eligible for initiation, which gradually cleared over time. Additionally, the monthly proportion of ART initiators with a known advanced HIV disease status decreased, although about a quarter of people still started ART with advanced HIV disease after increasing the CD4 eligibility for ART initiation to <500 cells/µL in January 2015 and during the UTT era. The pooled prevalence of advance HIV disease among ART-naïve patients from a systematic review of 53 studies conducted between 2010 and 2022 (the majority before 2016) was 43.4% (95% CI 40.1 to 46.8%). This pooled prevalence is consistent with the prevalence of advanced HIV disease in our study from April 2012 to January 2015, during which the CD4 count eligibility for ART initiation was <350 cells/µL. Additionally, our estimate of advance HIV disease in the UTT era is consistent with results from the studies that were conducted from 2016 onwards.^29 30^

We also found that TB prevalence among all initiators declined following the expansion of CD4 count eligibility criteria but remained stable among the subset of initiators with advanced HIV disease. Finally, among initiators without TB disease, the cumulative incidence of TB within 12 months of follow-up declined following UTT implementation, both in the whole cohort and in the cohort of individuals who initiated with advanced HIV disease. Generally, the impact of expanded ART access on the prevalence of advanced HIV disease at initiation, TB prevalence at initiation and TB incidence within 12 months of follow-up were similar by sex and age.

Some studies have previously assessed the impact of expanded initiation CD4 count eligibility on new ART initiations, advanced HIV disease and TB disease burden among people initiating and receiving ART from routine care settings.^914^,^16 18^ Results from a cohort study among adult participants in the rural Hlabisa HIV treatment programme in KwaZulu-Natal, South Africa, demonstrated increased new ART initiations in PLHIV but did not change among initiators with advanced HIV disease following the expansion of the initiation CD4 count eligibility from <200 to <350 cells/µL in August 2011.^15^ Other studies in South Africa have reported higher mean initiation CD4 count^8^ and a lower proportion of initiators with advanced HIV disease^9^ after UTT implementation than before. Additionally, the African cohort study in four countries reported increased new ART initiations at higher initiation CD4 counts following the implementation of policies and guidelines that expanded initiation CD4 count eligibility in the selected countries from 2006 to 2019.^31^

Regarding the impact of expanded ART access on TB burden, our results are consistent with a large-scale study in South Africa that showed a lower risk of recently diagnosed TB cases in communities with higher ART coverage.^18^ We found a cluster-randomised trial in Uganda that evaluated a UTT intervention involving population-level HIV testing and patient-centred linkage to ART versus population-level HIV testing in the control communities. At the end of the trial, the 1-year cumulative incidence of TB infection was 16% in the intervention and 22% in the control communities.^14^ Likewise, the risk of new TB infection was 27% lower with the UTT intervention.^14^ Another study investigated the impact of the UTT policy on TB incidence in a cohort of adults receiving ART in public health facilities in Ethiopia from 2014 to 2019.^16^ The study reported a TB incidence of 6.23 cases per 100 person-years before UTT implementation vs 2.10 cases per 100 person-years after UTT.^16^ Furthermore, estimates from WHO’s 2022 global TB report have shown a declining trend in the population-level incidence of TB co-burden in PLHIV in the WHO African Region and in South Africa^32^ after 2010.

We used large-scale data from routine ART clinics in a resource-limited setting. Therefore, our results are representative and relevant for ART programmes in such settings. The findings are reassuring, demonstrating the positive impact of expanded ART access in reducing the risk of advanced HIV disease and opportunistic co-infections, which could improve the health and well-being of PLHIV. Our findings indicate that extending the initiation CD4 count from <350 to <500 cells/µL in January 2015 allowed more PLHIV to start ART than previously. Later, removing the initiation CD4 count testing requirement and eligibility criteria with UTT implementation led to a further increase in new ART initiations. However, our results show that despite these gains, about 19.5% of PLHIV still presented with advanced HIV disease at initiation. This likely underestimates the prevalence of advanced HIV disease, as our definition was based solely on the CD4 count threshold. In the UTT era, about 16.5% of new ART initiators did not receive a CD4 count test, probably because it is no longer required for ART initiation, though it is still needed for routine monitoring of advanced HIV disease. People who initiate ART with advanced HIV disease in the UTT era may have missed the opportunity for early HIV diagnosis, encountered delayed access to ART initiation or disengaged from previous, undisclosed HIV care, highlighting bottlenecks in achieving the 95-95-95 UNAIDS 2025 HIV care targets.^33^ Our findings, therefore, emphasise the continuing urgency for health systems in resource-limited settings to prioritise access to routine HIV testing services, especially among higher-risk populations such as younger people, pregnant women and their male partners. This will help facilitate early HIV diagnosis and immediate linkage to care to exploit the full potential of UTT policy implementation.

Moreover, our study demonstrates the positive impact of expanded ART access on TB co-burden in PLHIV. Our findings suggest that people initiating ART in the UTT era are less likely to present with TB. This is due to more people initiating ART at higher CD4 counts with more robust immune function,^15 34 35^ reducing the risk of opportunistic co-infections such as TB.^36^,^38^ Our findings also indicate that people initiating ART without TB in the UTT era are less likely to develop new TB while on ART compared with the pre-UTT period. This could be attributed to early ART initiation,^15 34 35^ ensuring early viral suppression, which enhances consistent and prolonged immune function.^11 39 40^ Furthermore, we found a trend towards a decrease in TB incidence among initiators with advanced HIV disease. This suggests the positive impact of expanded ART access on TB transmission risk among PLHIV due to reduced exposure to the TB bacterium, as fewer people initiate ART with TB. We did not observe any impact of expanding the initiation CD4 count eligibility from <350 to <500 cells/µL in January 2015 on decreased TB disease incidence, but this could be due to the shorter duration of the criteria change (21 months) before UTT implementation in September 2016. It is therefore likely that the decreased TB incidence observed after UTT implementation represents the cumulative impact of the overall expanded ART access over time (including all initiation CD4 count criteria expansions).

Our study had some limitations. Although our data set was large, it came from only one province in South Africa and does not include hospital-based HIV care. We, therefore, acknowledge that outcomes could differ in other settings. Furthermore, we did not have pre-ART data, meaning people with AHD and TB who never started ART were not included, nor did we have data on TB preventative therapy trends, which could have affected the incidence of TB within 12 months. Also, data quality, correctness and completion rates in TIER.Net have improved over time compared with the early years after its implementation in 2010, which could bias our results.^41^ We also acknowledge the potential confounding of our study outcomes by changes in the clinic populations or other concurrent interventions that could have contributed to better outcomes in the UTT era, such as more efficacious and tolerable ART regimens and improvements in TPT.^42 43^ Therefore, participants who initiated ART recently or during the UTT era are more likely to have initiated more effective and tolerable regimens. Hence, they are more likely to be retained in care,^44^ virally suppressed^45 46^ and consequently less likely to develop new TB during ART. Moreover, the implementation of the Central Chronic Medication Dispensing and Distribution programme in KwaZulu-Natal in 2016 could have reduced patient volumes and, hence, reduced exposure to TB at the clinics, contributing to the declining TB burden we observed in the UTT era.

In conclusion, the expansion of the CD4 count eligibility for ART initiation over time has been associated with expanded access to ART and a healthier population of PLHIV with stronger immunity and a reduced risk of co-TB disease. Health systems in resource-limited and HIV-endemic settings should prioritise population-level early HIV diagnosis to maximise the full potential of UTT policy implementation, while continuing CD4 count testing to diagnose AHD. WHO’s recommended package of care for people with advanced HIV disease, which includes CD4 count monitoring, TPT and prophylaxis for other opportunistic infections, rapid ART initiation and intensified adherence support, should also be a core component of ART programmes in endemic countries.^47^

## Data Availability

Data may be obtained from a third party and are not publicly available.
